# Study on the structural, optical and magnetic properties of cobalt-doped CdSSe quantum dots

**DOI:** 10.1039/d6ra00412a

**Published:** 2026-04-08

**Authors:** N. T. T. Hoan, N. T. Tung, N. T. T. Hang, D. T. Linh, N. V. Truong, N. X. Ca, V. T. K. Lien, D. T. Hue, N. T. H. Nga, P. V. Duong, P. M. Tan

**Affiliations:** a Faculty of Fundamental and Applied Sciences, Thai Nguyen University of Technology Vietnam; b Institute of Science and Technology, Thai Nguyen University of Sciences Vietnam canx@tnus.edu.vn; c Institute of Theoretical and Applied Research Hanoi, Duy Tan University Vietnam; d Faculty of Physics, Thai Nguyen University of Education Vietnam; e Faculty of Mechanical Engineering, Thuyloi University Vietnam; f Institute of Physics, Vietnam Acedemy of Science and Technology Vietnam; g Faculty of Fundamental Sciences, Posts and Telecommunications Institute of Technology Vietnam tanpm@ptit.edu.vn

## Abstract

Co-doped CdSSe alloy quantum dots (QDs) were successfully synthesized *via* a wet chemical hot-injection method, with Co^2+^ doping concentrations ranging from 1 to 10% at. In addition to the intrinsic band gap tunability of CdSSe QDs, Co incorporation introduces magnetic functionality, enabling the development of diluted magnetic semiconductor nanostructures for optoelectronic and spintronic applications. The effects of sulfur/selenium ratio and cobalt doping on the structural, optical, photoluminescence, and magnetic properties of CdSSe QDs were systematically investigated. X-ray diffraction results confirm that both undoped and Co-doped CdSSe QDs crystallize in the cubic zinc blende structure, with no secondary phases detected. The lattice constant decreases with increasing Co concentration due to the substitution of smaller Co^2+^ ions for Cd^2+^ ions in the host lattice. Optical absorption and photoluminescence measurements reveal that the emission wavelength of CdS_*x*_Se_1−*x*_ QDs can be effectively tuned across the visible region by adjusting the S/Se ratio. Upon Co^2+^ doping, a pronounced blue shift of both absorption and photoluminescence peaks is observed, accompanied by an increase in band gap energy, indicating strong modification of the electronic structure induced by Co-related energy levels. This behavior is attributed to the substitution of Cd^2+^ by smaller Co^2+^ ions, which induces compressive lattice strain and shifts the conduction band edge to higher energy. Time-resolved photoluminescence analysis shows a decrease in carrier lifetime with increasing Co concentration, attributed to enhanced non-radiative recombination *via* Co^2+^-induced trap states. Magnetic measurements demonstrate that Co-doped CdSSe QDs exhibit weak room-temperature ferromagnetism coexisting with diamagnetic behavior, with saturation magnetization increasing up to 5% Co doping and decreasing at higher concentrations due to the onset of antiferromagnetic interactions. These results demonstrate that Co doping is an effective method for simultaneously tuning the optical and magnetic properties of CdSSe QDs, making them promising candidates for optoelectronic and spintronic applications.

## Abstract 2

EDX elemental mapping of CdSSe doped Co 3% for cadmium, sulfur, selenium and cobalt; the corresponding EDX spectra of the as-synthesized CdSSe doped 3%.

## Graphical abstract

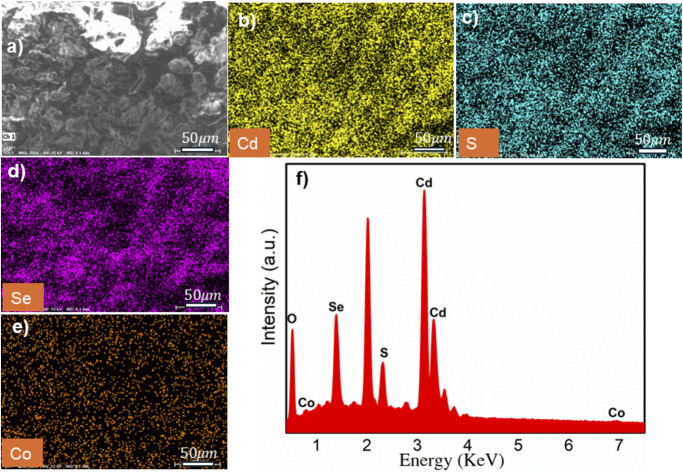

## 1 Introduction

Semiconductor quantum dots (QDs) have attracted great attention from scientists because of their special properties. The shape, crystal structure, and optical properties of QDs depend heavily on the fabrication conditions and particle size. QDs exhibit a strong quantum confinement effect due to their very small size, typically ranging from 2 to 10 nm. Therefore, QDs have unique optical and electrical properties, suitable for applications in solar cells,^1,2^ light-emitting diodes,^3,4^ photocatalysis,^5,6^ and electronics.^7^ Each application requires different particle sizes to produce emission spectra at appropriate wavelengths. The solution to change the emission wavelength while maintaining a small size is to use alloyed QDs. Among semiconductor QDs, ternary alloy CdSSe QDs are widely studied due to their tunable bandgap and documented stability. By changing the S/Se ratio, the emission wavelength of CdSSe QDs can be tuned within the visible light region.^8^ This makes them suitable for applications in optoelectronic devices, solar cells, and biomedical imaging.^1–7^

In recent decades, to create new materials possessing both optical and electromagnetic properties, semiconductor QDs are often doped with transition metals. These transition metal-doped semiconductor QDs exhibit unique and superior properties compared to undoped semiconductor QDs. Transition metals create energy traps and act as luminescent centers, thereby strongly affecting the optical properties of QDs. Transition metal-doped QDs also exhibit a large Stokes shift, which helps minimize self-absorption and energy transfer, while significantly enhancing thermal and chemical stability, as well as prolonging luminescence lifetime. Many transition metal ions, including Mn^2+^, Cu^2+^, Ni^2+^, Co^2+^, and Ag^+^, have been doped into semiconductor QDs, making these QDs attractive for various applications. Studies have indicated that energy levels within the forbidden gap exist when doping transition metal ions into semiconductor QDs. Interestingly, without needing to change the size, shape, or create complex nanostructures, transition metals significantly alter the emission wavelength of QDs. P. Elavarthi *et al.* investigated the effect of Cr doping concentration on the optical properties of CdS, observing that Cr doping transformed the emission from a strong violet band in undoped CdS nanoparticles to a broad emission band in CdS:Cr nanoparticles.^9^ As Cr concentration increases, the emission wavelength changes, and the emission intensity increases compared to the CdS host lattice. Cu doping concentration strongly affects the optical properties of CdTeSe QDs. At low Cu concentration (*x* = 0.005), the PL spectrum exhibits two emission peaks: (i) an excitonic emission peak (∼644 nm) and (ii) an emission peak from lattice defects, Cu^2+^ dopant (∼820 nm). When the Cu concentration is increased (*x* = 0.05), only the long-wavelength emission peak remains, indicating that the Cu^2+^related luminescence centers are dominant.^10^ Furthermore, doping with transition metals also imparts both optical and magnetic properties to the QDs, making them suitable for applications in the field of spintronics. L. Saravanan *et al.* synthesized cobalt-doped CdSe nanocrystals *via* an aqueous chemical co-precipitation method.^11^ The emission peak at 515 nm (for CdS:Co 2%) exhibits a blue shift as the Co doping level increases to 4% and 6%. A pronounced decrease in emission intensity is observed for the 6% Co sample, attributed to dopant ions in the host lattice acting as nonradiative recombination centers. However, at room temperature, the CdS samples doped with 4% and 6% Co exhibit clear ferromagnetic hysteresis loops, with the highest saturation magnetization observed for CdS:Co (6%), confirming the formation of a diluted magnetic semiconductor (DMS). K.A. Bogle *et al.* synthesized Co-doped CdS using a radiation synthesis method.^12^ The authors focused on investigating the formation of dopant-defect complexes and their influence on the electronic structure of the QDs. Meanwhile, CdS:Co QDs exhibit weak ferromagnetism, with magnetization increasing as the Co concentration rises from 1% to 3%, followed by a decrease at 5% Co. In contrast, Hu *et al.* reported relatively strong room-temperature ferromagnetism in Co-doped CdS nanocrystals.^13^ In these studies, the authors consistently concluded that cobalt is incorporated into the host lattice as Co^2+^ ions rather than as metallic clusters or separate Co nanoparticles. Specifically, Co^2+^ ions substitute for Cd^2+^ sites within the crystal lattice. The absence of diffraction peaks associated with Co or secondary phases (such as CoO or CoS) in the XRD patterns provides experimental evidence supporting this substitution mechanism.^11–13^ Binary QDs such as Co-doped CdS and CdSe have been extensively investigated; however, their optical properties are primarily tuned through particle size control based on the quantum confinement effect. Synthesizing Co-doped CdSSe alloy QDs enables versatile modulation of the bandgap energy (1.7–2.4 eV) through stoichiometry adjustment, offering an additional degree of freedom beyond size-dependent tuning and yielding a multifunctional material with dual optical and magnetic characteristics. When Co^2+^ ions are introduced into the CdSSe lattice, they can substitute for Cd^2+^ sites and introduce localized 3d electronic states within the host semiconductor matrix. The presence of these magnetic ions gives rise to sp–d exchange interactions between the band carriers (electrons and holes) and the localized d-electrons of Co^2+^. Such interactions can modify the electronic structure and carrier recombination dynamics of the host material. In addition, Co-related impurity levels may act as carrier trapping centers, influencing the radiative and non-radiative recombination processes and consequently affecting the optical properties of CdSSe quantum dots.

However, the successful synthesis of doped QDs with controlled composition and dopant distribution remains a challenge. Accurate control of dopant concentration needs to be optimized in order to achieve the desired structure and properties. These properties are significantly influenced by fabrication conditions such as reaction temperature, synthesis time, ligand concentration, precursor ratio, *etc.* In this study, we will investigate the effect of Co concentration on the structural characteristics, optical properties, and magnetic properties of Co-doped CdSSe QDs.

## 2 Experimental

### 2.1 Materials

The chemicals used to fabricate CdSSe QDs and Co-doped CdSSe QDs include: CdO powder (99.5%), Se powder (99, 98%), S powder (99, 98%), cobalt(ii) nitrate hexahydrate (Co(NO_3_)_2_·6H_2_O, 98%), 1-octadecene (ODE, 90%), trioctylphosphine (TOP, 97%), axit oleic (OA, 90%), *n*-hexane và isopropanol (98%). All chemicals were purchased from Sigma-Aldrich.

### 2.2 Synthesis of CdSSe QDs

CdSSe QDs were synthesized by the wet chemical method. S (Sulfur) powder and Se (Selenium) powder were stirred in ODE and TOP solvents at 100 °C until the precursors completely dissolved, yielding a solution containing S^2−^ và Se^2−^. Depending on the desired S/Se ratio, the amounts of S and Se precursors in the samples vary. CdO was stirred with OA and ODE in a three-neck flask at 200 °C for 50 minutes to obtain an orange-yellow solution containing Cd^2+^ ions. S^2−^ and Se^2−^ containing solutions were injected into the Cd^2+^ containing solution, and the reaction was maintained for 60 minutes, yielding a solution containing CdSSe QDs. The QDs were then purified by centrifugation with isopropanol and redispersed in *n*-hexane for subsequent measurements. The reactions were carried out in a N_2_ gas atmosphere to prevent oxidation.

### 2.3 Synthesis of Co-doped CdSSe QDs

**Fig. 1:**
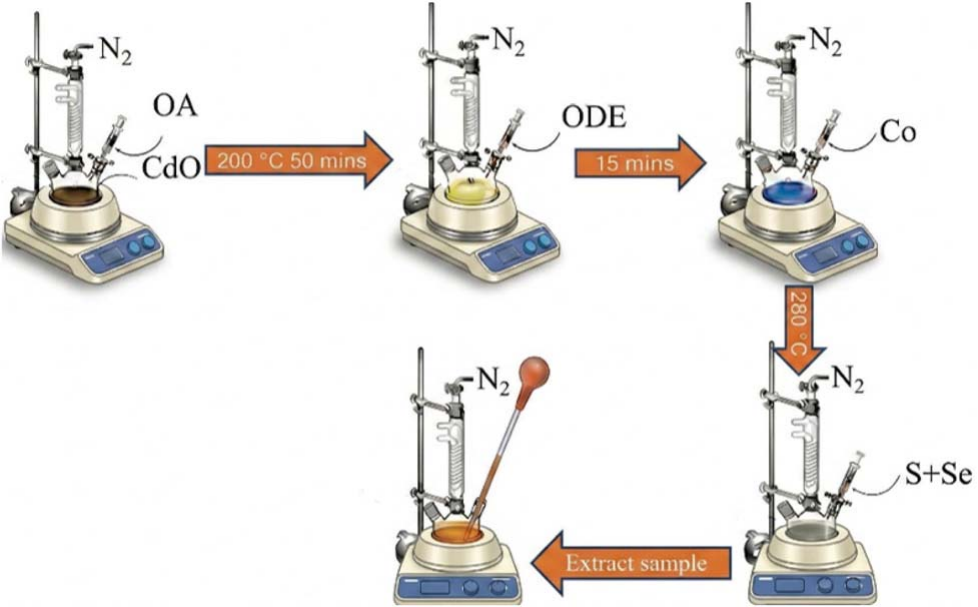
Synthesis scheme of CdSSe:Co quantum dots.

Co-doped CdSSe QDs were fabricated using CdO, Co(NO_3_)_2_·6H_2_O, Se, and S precursors. The Cd^2+^, S^2−^, and Se^2−^ solutions were prepared similarly to the synthesis of CdSSe QDs. Co(NO_3_)_2_·6H_2_O was stirred with TOP and ODE at 100 °C for 60 minutes yielded a characteristic cobalt blue solution containing Co^2+^ ions. The amount of Co (NO_3_)_2_·6H_2_O was adjusted based on the desired Co/Cd ratio. The solution containing Co^2+^ ions was injected into the solution containing Cd^2+^ ions. After that, the solution containing S^2−^ and Se^2−^ was swiftly injected into a three-neck flask at 280 °C, and the reaction was maintained for 60 minutes, yielding CdSSe:Co QDs. The CdSSe:Co QDs were cooled to room temperature, and the subsequent process was the same as for CdSSe QDs. The synthesis scheme of CdSSe and CdSSe:Co QDs is observed in Fig. 1.

### 2.4 Characterization

The crystal structure of the samples was analyzed using an X-ray diffractometer (Bruker D8-Advance) using Cu-Kα radiation (*λ* = 1.5406 Å). TEM images were obtained using a JEOL JEM-1400 transmission electron microscope operated at 120 kV. The surface morphology and microstructural features were examined by field-emission scanning electron microscopy (FESEM) using a Regulus 8100 microscope. Elemental composition and distribution were determined with an XFlash 6130 energy-dispersive X-ray (EDX) detector integrated into the FESEM system. Ultraviolet-visible (UV-vis) absorption spectra were recorded on a Jasco V-770. Photoluminescence (PL) spectra and fluorescence lifetimes were measured using an FLS1000 fluorescence spectrometer. The emission spectra were recorded in the wavelength range of 390–900 nm with a step size of 1 nm, and the excitation wavelength was fixed at 375 nm.

## 3 Results and discussion

### 3.1 Effect of S/Se ratio on the optical properties, crystalline structure of the CdS_*x*_Se_1−*x*_ QDs

**Fig. 2:**
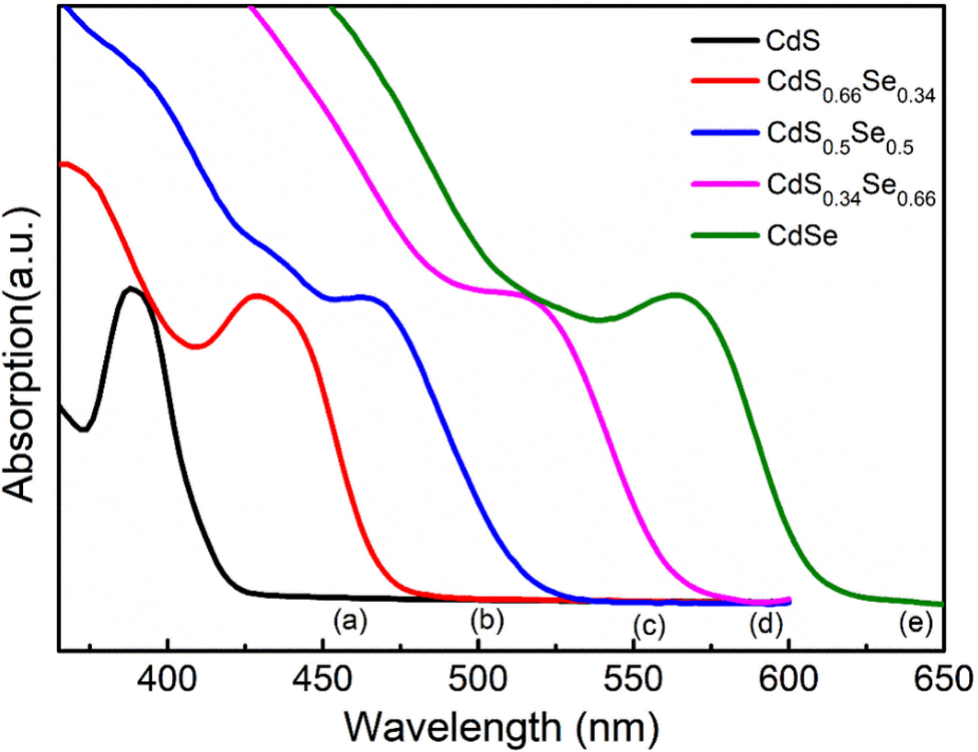
UV-vis absorption (Abs) of CdS_*x*_Se_1−*x*_: (a) *x* = 1, (b) *x* = 0.66, (c) *x* = 0.5, (d) *x* = 0.34, (e) *x* = 0.

**Fig. 3:**
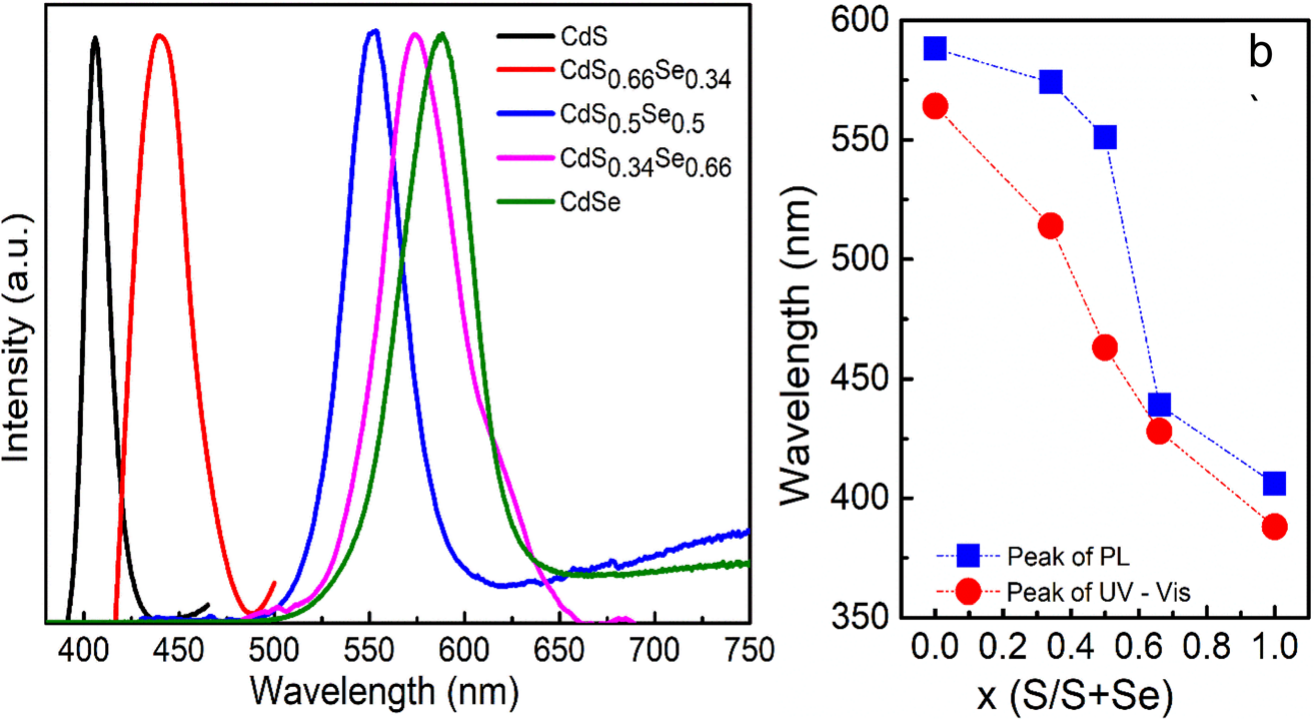
(a) PL spectra, (b) the variation of Abs and PL peaks in relation to the composition ratio, *x*, of CdS_*x*_Se_1−*x*_ alloy QDs.

In this study, CdS_*x*_Se_1−*x*_ QDs with different *x* ratios (*x* = 1, 0.66, 0.5, 0.34, 0) were synthesized using a wet chemical method. The optical properties of CdS_*x*_Se_1−*x*_ QDs are significantly influenced by the S/Se ratio. To investigate the effect of the S/Se ratio on the optical properties of CdS_*x*_Se_1−*x*_ QDs, the absorption and PL spectra of the samples were measured and are presented in Fig. 2 and 3.

Fig. 2 shows that the absorption peaks of the samples shift towards longer wavelengths as *x* gradually decreases (Se content increases) (this is consistent with previous studies.^14,15^ Specifically, the CdS (*x* = 1) sample has an absorption peak around 388 nm, while the CdSe (*x* = 0) sample has an absorption peak at 564 nm. Intermediate samples such as CdS_0.66_ Se_0.34_, CdS_0.5_Se_0.5_, and CdS_0.34_ Se_0.66_ exhibit absorption peaks at approximately 429 nm, 468 nm, and 515 nm, respectively. The red shift of the absorption spectrum as *x* decreases (increasing CdSe content) primarily originates from the chemical composition, reducing the band gap of the QD. The bulk band gaps of CdS and CdSe are 2.4 eV and 1.7 eV, respectively. CdSe has a lower band gap, so increasing the CdSe content shifts the absorption peak to longer wavelengths. The absorption peaks of CdS_0.66_Se_0.34_, CdS_0.5_Se_0.5_, and CdS_0.34_ Se_0.66_ lie between the absorption peaks of CdS and CdSe, demonstrating the formation of CdSSe QDs, instead of the formation of two-component CdS and CdSe QDs.

The photoluminescence (PL) spectra of CdS_*x*_Se_1−*x*_ QDs with different S/Se ratios are shown in Fig. 3a. The results reveal that the QDs exhibit sharp and narrow emission peaks, indicating a relatively uniform particle size distribution. Specifically, as sulfur content (*x*) gradually decreases (meaning the selenium ratio increases), the excitonic emission peak shifts from blue (410 nm for CdS QDs) to red (610 nm for CdSe QDs).^16^ This red shift indicates a gradual decrease in the band gap energy (*E*_g_) of CdS_*x*_Se_1−*x*_ alloys as the composition changes from S-rich to Se-rich CdSSe QDs. This is consistent with the *E*_g_ (band gap) calculated from the absorption spectrum: as *x* decreases from 1 to 0, the *E*_g_ decreases correspondingly from 3.2 eV to 2.2 eV (Table 1). This result indicates that the emission wavelength of CdS_*x*_Se_1−*x*_ QDs can be tuned by adjusting the S/Se ratio in the alloy structure. Fig. 3b illustrates the dependence of the excitonic absorption peak and the excitonic PL peak on the composition *x*. Both the blue and red curves in Fig. 3b show a red shift of the spectral peak as *x* decreases. The photoluminescence peak is always located at a longer wavelength than the corresponding absorption peak, consistent with the Stokes shift.

**Table 1:** Lattice parameters, crystallite size and bandgap energy of CdS_*x*_Se_1−*x*_

Sample	2*θ* (°)	*a* (Å)	*β* (°)	*D* (nm)	*δ* (nm^−2^)	*E*_g_ (eV)
CdS	26.61	5.79	5.12	1.5	0.43	3.2
CdS_0.66_Se_0.34_	26.07	5.85	5.13	1.51	0.44	2.9
CdS_0.5_Se_0.5_	25.04	5.93	3.95	1.96	0.26	2.49
CdS_0.34_Se_0.66_	24.38	6.01	4.65	1.66	0.35	2.41
CdSe	24.31	6.05	5.99	1.29	0.59	2.2

**Fig. 4:**
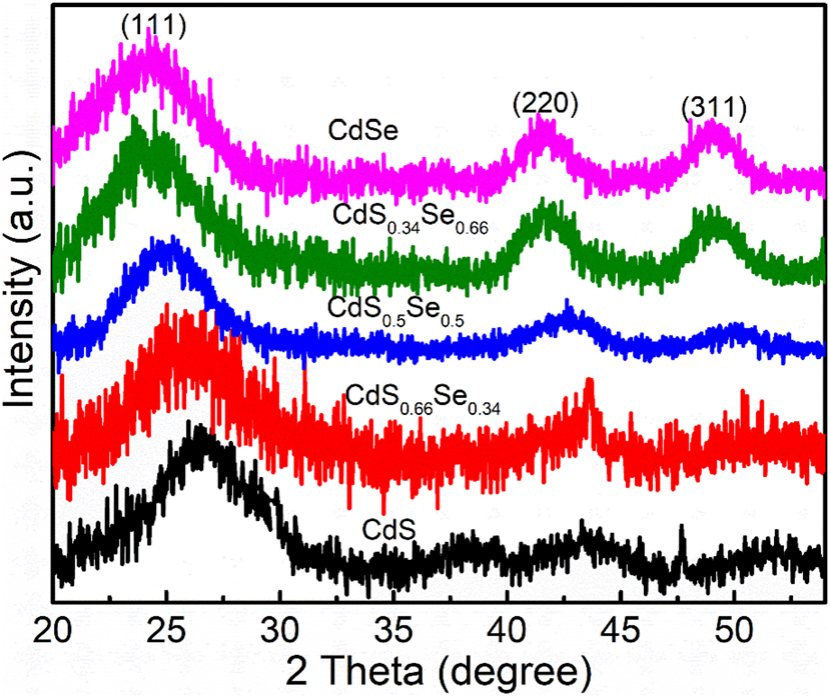
XRD patterns of CdS_*x*_Se_1−*x*_ alloy QDs.

Fig. 4 presents the X-ray diffraction (XRD) patterns of CdS_*x*_Se_1−*x*_ QDs. XRD patterns show that all samples have three characteristic diffraction peaks corresponding to the crystal planes (111), (220) and (311) of the zinc blende (ZB).^14^ This demonstrates that the S/Se ratio does not change the ZB structure of CdS_*x*_Se_1−*x*_. The positions of these diffraction peaks all shift towards smaller 2*θ* angles as the value of *x* changes from 1 to 0, due to the increase in lattice constant when S atoms are replaced by Se^14^ (This has also been observed in CdTe_*x*_Se_1−*x*_^17^). This shift indicates that the alloy composition of CdS_*x*_Se_1−*x*_ QDs has been tuned according to the S/Se ratio, from S-rich QDs to Se-rich QDs.

The CdS_*x*_Se_1−*x*_ QDs have a cubic structure, so the lattice constant ‘*a*’ is determined by the formula:^18,19^1
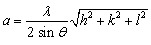
where *θ* is the Bragg diffraction angle, *λ* is the X-ray wavelength (*λ* = 1.54 Å), and *h*, *k*, *l* are the Miller indices of the crystal plane.

Using the (111) lattice plane, the lattice constants *a* of CdS_*x*_Se_1−*x*_ were calculated to be approximately 5.79 Å, 5.85 Å, 5.93 Å, 6.01 Å, and 6.05 Å for *x* = 1, *x* = 0.66, *x* = 0.5, *x* = 0.34, and *x* = 0, respectively, as shown in Table 1. The S/(S+Se) ratio (*x*) and the lattice constant (*a*) have a linear relationship, indicating the formation of CdS_*x*_Se_1−*x*_ alloy. To calculate the average crystallite size of the particles from the XRD spectrum, the Debye–Scherrer equation is used:^20^2
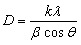
Here: *D* is the crystallite size, *k* = 0.9 is a constant, *λ* is the X-ray wavelength, *θ* is the Bragg angle, and *β* is the width at half maximum (FWHM) of the diffraction peak. By substituting the values, we calculated the average crystallite size of the CdS_*x*_Se_1−*x*_ QDs to be 1.5 nm, 1.51 nm, 1.96 nm, 1.66 nm, and 1.29 nm, corresponding to *x* values of 1, 0.66, 0.5, 0.34, and 0, respectively. Since the crystallite size remains nearly unchanged, the pronounced shifts observed in the absorption spectrum (Fig. 2) and photoluminescence spectrum (Fig. 3) are primarily attributed to the variation in chemical composition of the QDs. Thus, the synthesis and analysis results demonstrate that controlling the S/Se ratio is an effective approach to tailoring the crystal structure and optical properties of CdSSe QDs.

**Fig. 5:**
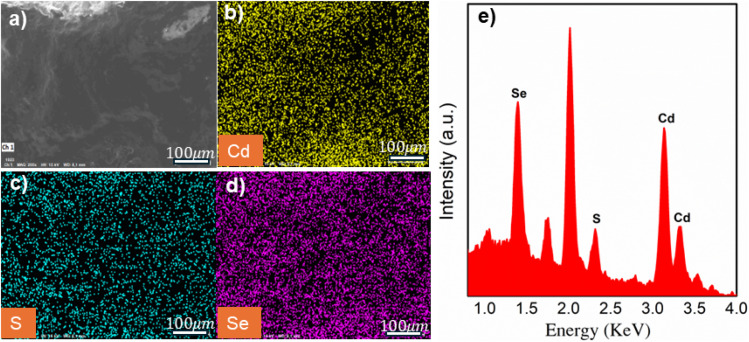
(a) EDX elemental mapping of CdS_0.5_Se_0.5_ for (b) cadmium, (c) sulfur, and (d) selenium. (e) The corresponding EDX spectra of the as-synthesized CdS_0.5_Se_0.5_.

To determine the elemental composition of CdS_0.5_Se_0.5_ QDs, we performed EDX spectroscopy measurements and EDX mapping (Fig. 5). EDX mapping (Fig. 5a–d) showed that the elements Cd, S, and Se were homogeneously distributed throughout the sample, demonstrating the uniform dispersion of the QDs. EDX spectrum analysis revealed that in CdSSe QDs, the composition of the elements Cd, S, and Se were 46.23%, 23.57%, and 30.2%, respectively (Fig. 5e). The S/Se ratio measured in the experiment was 1 : 1, whereas the actual S/Se ratio determined from the EDX spectrum was 0.78 : 1. This indicates that the chemical activity of Se is stronger than that of S. The clear presence of Cd, Se, and S elements in the EDX spectrum, together with their uniform distribution, confirms that CdSSe QDs have been successfully synthesized with homogeneous dispersion.

### 3.2 Impact of Co^2+^ ions on the structural and optical properties of CdS_*x*_Se_1−*x*_ QDs

CdS_0.5_Se_0.5_ QDs (hereafter referred to as CdSSe) were selected for Co^2+^ doping with concentrations of 1, 3, 5, and 10%, respectively.

#### 3.2.1 X-ray diffraction pattern

**Fig. 6:**
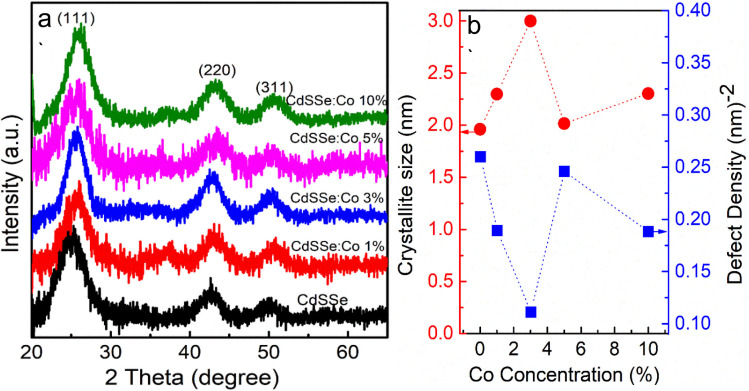
(a) X-ray diffraction (XRD) patterns of pure and 1–10% Co-doped CdSSe. (b) The dependence of crystallite size and defect density on Co concentration.

The structure, lattice constant, and crystallite size of Co-doped CdSSe QDs with different concentrations were analyzed by X-ray diffraction (XRD) patterns (Fig. 6a). Fig. 6a shows that all obtained samples have three main diffraction peaks at 2*θ* angles around 25°, 42°, and 50°, corresponding to the (111), (220), and (311) crystal planes of the cubic zinc blende structure. With increasing Co content, the diffraction peaks shift toward higher 2*θ* angles (from 25° to 26° for (111) crystal plane). Since CdSSe:Co QDs have a cubic structure, the lattice constant (a), calculated from eqn (1), is 5.93, 5.90, 5.86, 5.81, and 5.80 nm corresponding to Co^2+^ concentrations of 0, 1, 3, 5, and 10%, respectively. Thus, as the Co^2+^ concentration increases, the lattice constant of the samples decreases. This can be attributed to the substitution of smaller Co^2+^ ions (0.74 Å) for larger Cd^2+^ ions (0.96 Å) in the crystal lattice, leading to lattice contraction.^21–23^ The absence of additional diffraction peaks beyond the main crystal planes indicates that the doped material maintains its characteristic zinc blende structure without forming secondary phases or new cobalt-containing compounds. This proves that Co^2+^ has been successfully doped into the CdSSe lattice *via* a substitutional mechanism.

Using eqn (2), the average crystallite size of the samples was calculated to be in the range of 1.96–3 nm (Table 2). This indicates that Co^2+^ doping does not significantly alter the crystallite size of CdSSe QDs. Fig. 6b illustrates the variation of crystallite size (*D* (nm) = 0.9*λ*/*β* cos *θ*) and defect density (*δ* (nm^−2^) = 1/*D*^2^) of Co-doped CdSSe QDs as a function of Co doping concentration. The crystallite size and defect density of the samples are *D* = 1.96, *δ* = 0.26 for CdSSe, *D* = 2.29, *δ* = 0.19 for CdSSe:Co 1%, *D* = 3, *δ* = 0.11 for CdSSe:Co 3%, *D* = 2.02, *δ* = 0.24 for CdSSe:Co 5%, *D* = 2.3, *δ* = 0.18 for CdSSe:Co 10%. Thus, the CdSSe:Co 3% sample exhibits the largest crystallite size and the lowest defect density.

**Table 2:** Lattice parameters, crystallite size and bandgap energy of CdSSe doped Co

Sample	2*θ* (°)	*a* (Å)	*β* (°)	*D* (nm)	*δ* (nm^−2^)
CdSSe	25.04	5.93	3.95	1.96	0.26
CdSSe:Co 1%	25.67	5.90	3.36	2.29	0.19
CdSSe:Co 3%	25.72	5.86	2.58	3.0	0.11
CdSSe:Co 5%	25.82	5.81	3.84	2.02	0.24
CdSSe:Co 10%	25.97	5.80	3.36	2.30	0.18

**Fig. 7:**
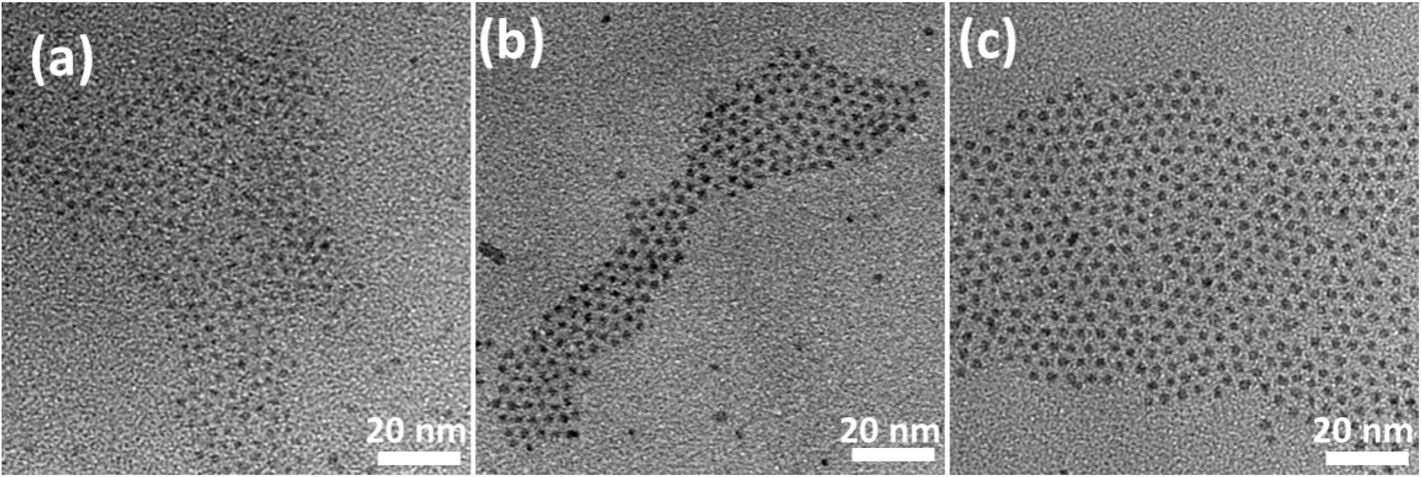
TEM images of (a) CdSSe pure, (b) CdSSe:Cu 5% and (c) CdSSe:Cu 10% QDs.

TEM images of undoped CdSSe, 3% Co-doped CdSSe, and 10% Co-doped CdSSe QDs are illustrated in Fig. 7. Fig. 7 reveals that all samples consist of spherical particles with a narrow size distribution. The average diameters are approximately 2.5 nm for the undoped CdSSe, 3.6 nm for the 3% Co-doped CdSSe, and 4.1 nm for the 10% Co-doped CdSSe. These results demonstrate that the size of the QDs increases with increasing Co doping concentration. The increase in particle size with increasing Co concentration can be attributed to the enhanced crystal growth kinetics induced by Co incorporation. The substitution of Cd^2+^ by Co^2+^ modifies the local lattice environment and reduces surface energy, thereby promoting particle coalescence and grain growth. Additionally, higher dopant concentration may facilitate Ostwald ripening during synthesis, leading to the formation of larger QDs.

#### 3.2.2 Absorption and photoluminescence properties

**Fig. 8:**
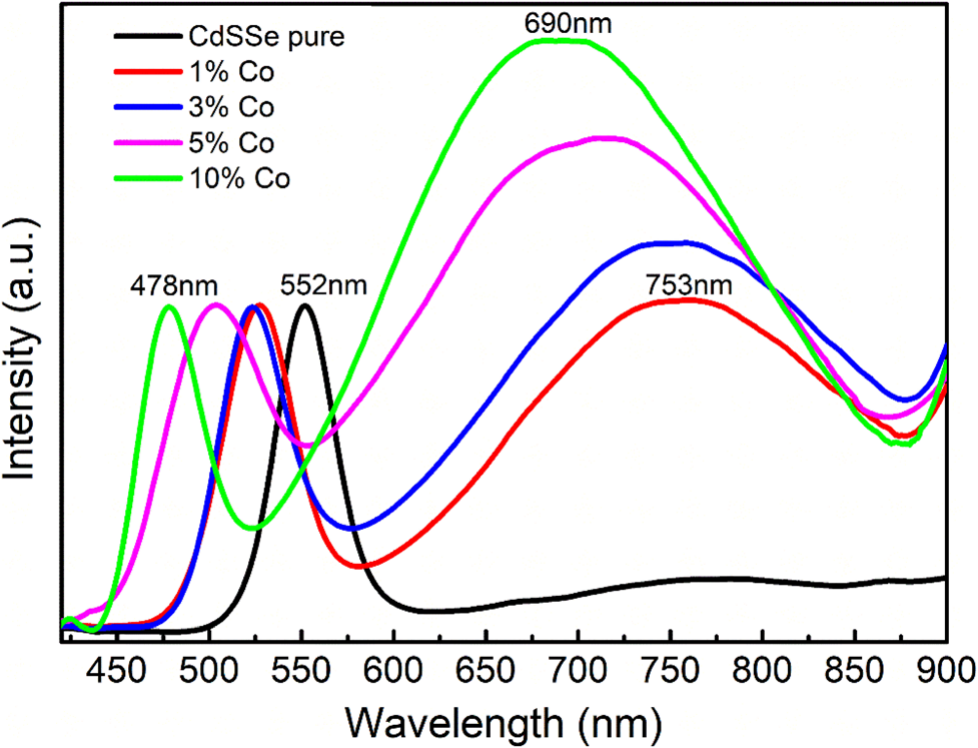
PL spectra of CdSSe doped Co.

To investigate the optical properties of CdSSe:Co QDs, PL spectra and UV-Vis spectra were measured. Fig. 8 presents the PL spectra of CdSSe QDs and Co-doped CdSSe QDs (1, 3, 5, and 10% Co), recorded at room temperature under an excitation wavelength of 375 nm. Fig. 8 shows two emission peaks in the PL spectra: one located in the shorter wavelength region and another in the longer wavelength region. For CdSSe QDs, a well-defined emission peak can be clearly observed at around 552 nm, which corresponds to the yellow emission region. This emission peak is attributed to excitonic recombination, where electrons in the conduction band recombine with holes in the valence band of CdSe. Almost no long-wavelength emission (emission related to surface states) was observed in the PL spectra of CdSe QDs, which indicates that the surface of the CdSSe QDs was well passivated by ligands such as OA and TOP. PL spectrum analysis showed that the exciton emission peak of Co-doped CdSSe QDs (0, 1, 3, 5, 10% Co) tended to blue-shift towards shorter wavelengths from 552 nm to 478 nm (corresponding to an increase in emission energy from 2.25 eV to 2.59 eV).^24^ This blue shift is attributed to Co^2+^ doping, altering the radiative recombination mechanism of the host lattice. When incorporated into CdSSe QDs, Co^2+^ creates energy traps within the band gap of the host material. As the Co^2+^ doping concentration increases, these traps tend to shift towards the valence band. This leads to the recombination between free electrons in energy traps created by Co^2+^ and holes in the valence band, resulting in a blue shift of the fluorescence spectrum.^25^ The blue shift trend of the excitonic emission peak with increasing concentration (from 552 nm to 478 nm) in the present study parallels observations in the ZnS:Co system synthesized by Salem *et al.*, where the PL emission shifted from 360 nm to 349 nm as the Co content rose from 0 to 1%.^26^ However, Abdulwahab *et al.*, in their study of Co-doped CdSe thin films, demonstrated that the bandgap energy of CdSe:Co decreases as the Co doping concentration increases.^27^ This redshift is attributed to the incorporation of Co into the CdSe host lattice, which creates a high density of point defects such as interstitials, antisite defects, and vacancies.

The photoluminescence spectrum also shows a broad emission peak towards longer wavelengths at approximately 690 nm. This emission peak may originate from surface states, lattice defects, or impurity-related emissions. Such emissions usually exhibit a broad spectral width due to the wide energy distribution of the emitting states. Co affects the optical properties of CdSSe QDs because this ion has the ability to create new energy levels within the band gap of the material. In the CdSSe crystal lattice, Co^2+^ ions tend to substitute Cd^2+^ sites, since the bonding energy of S and Se atoms is generally higher than that of Cd.^24^ Partial substitution of Cd^2+^ ions with Co^2+^ can introduce lattice defects and modify the band structure. These changes may result in a broader photoluminescence spectrum due to the formation of trap states and localized energy levels within the bandgap.^25^ Furthermore, the long-wavelength emission peak significantly increased in intensity as the Co^2+^ doping concentration increased from 0 to 10%. This enhancement is attributed to the formation of Co^2+^-related energy levels within the band gap, which act as impurity-assisted radiative recombination centers in the quantum dot structure. This result once again demonstrates that Co^2+^ ions have been incorporated into the CdSSe host lattice and have replaced the position of Cd^2+^ ions. However, F. Ibraheem suggests that the intensity of the emission peaks tends to decrease gradually with increasing Co^2+^ ion concentration.^29^ This decrease is due to the more effective suppression of surface traps by the doping process. Another reason is the increase in non-radiative relaxation processes in the QDs as the Co^2+^ doping concentration increases.

**Fig. 9:**
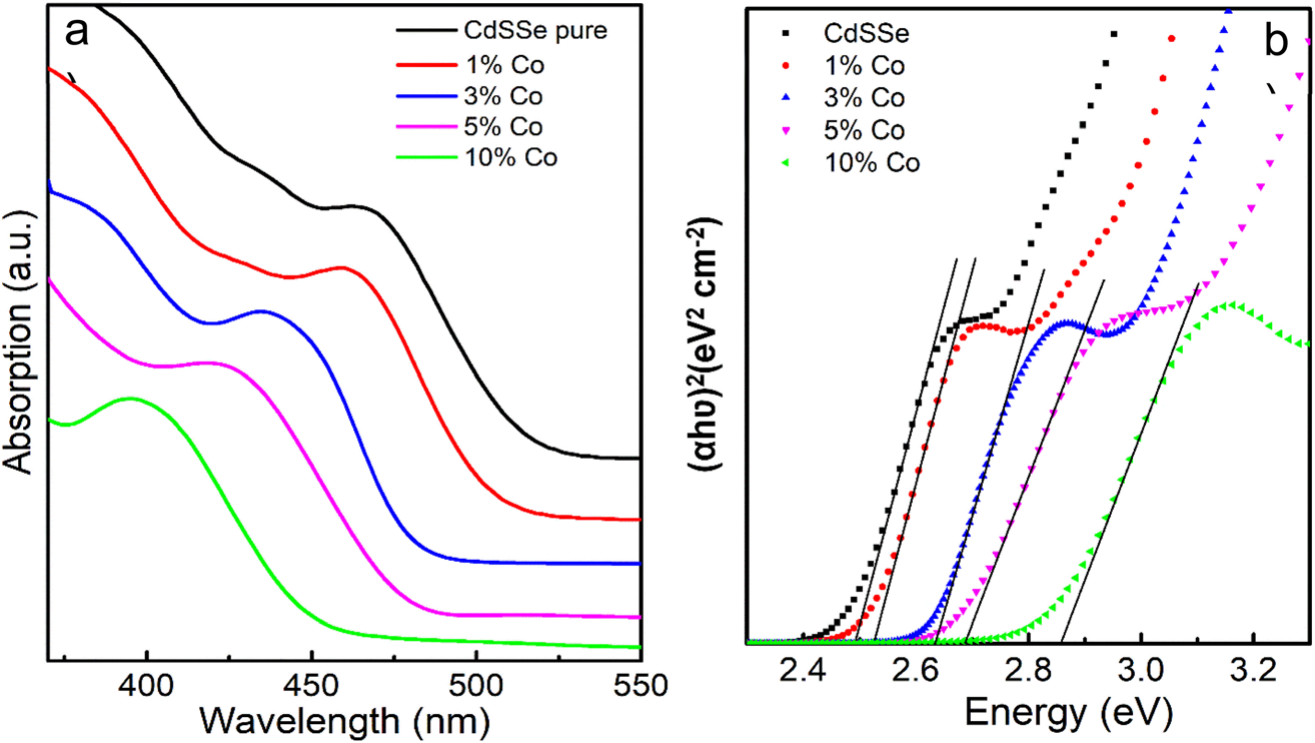
(a) Plot of absorption spectra of pure, 1%, 3%, 5% and 10% Co doped CdSSe QDs. (b) The Tauc plot for (*αhν*)^2^*vs.* (*hν*) of Co doped CdSSe QDs.

Fig. 9a shows the absorption spectra of 5 samples: CdSSe, CdSSe:Co 1%, CdSSe:Co 3%, CdSSe:Co 5%, CdSSe:Co 10%. The band gap energy of bulk CdS_*x*_Se_1−*x*_ alloy semiconductors can be estimated using the Bowing equation, which describes the nonlinear relationship between the band gap energy and the chemical composition of the alloy:^28,29^

3 *E*_g_(*x*) = *xE*_g_(CdS) + (1 − *x*)*E*_g_(CdSe) − *bx*(1 − *x*)

where *E*_g_(*x*) is the band gap energy of the bulk semiconductor alloy CdS_*x*_Se_1−*x*_ (*x* = 0.5), *E*_g_(CdS) = 2.42 eV and *E*_g_(CdSe) = 1.74 eV are, respectively, the band gap energies of pure bulk CdS and CdSe, and *b* is the bowing parameter, which defines the extent of nonlinearity in the variation of band gaps with composition. For bulk CdSSe alloy semiconductors, *b* = 0.29.^30,31^ From eqn (3), the band gap energy of bulk CdSSe is calculated to be 2.01 eV. Fig. 9a and b show that all absorption peaks of the samples are located in a higher energy region compared to bulk CdSSe at 616 nm (2.01 eV). This indicates that the obtained particles are of nanometer size. The band gap energy of undoped CdSSe QDs and Co^2+^ doped CdSSe QDs was determined by the Tauc plot in Fig. 9b. Specifically, the band gap (*E*_g_) of the QDs was determined using the following equation:

4 (*αhυ*) = *A*(*hυ* − *E*_g_)^1/2^

where *α* is the absorption coefficient, *E*_g_ is the band gap of the QDs, and *A* is a constant. Fig. 9b shows the plot of (*αhν*)^2^*versus* photon energy (*hν*) for undoped and Co-doped CdS QDs. By extrapolating the linear portion of the curve to the *hν* axis, the band gap energy (*E*_g_) was determined. As the Co^2+^ concentration increases in the doped samples, the absorption peaks tend to blue shift,^28^ as illustrated in Fig. 9a. Specifically, the absorption peak shifts from 468 nm (2.49 eV) for CdSSe QDs to 461 nm (2.52 eV) for 1% Co-doped CdSSe, 437 nm (2.63 eV) for 3% Co, 423 nm (2.69 eV) for 5% Co, and 395 nm (2.84 eV) for 10% Co-doped CdSSe QDs (Table 3). Thus, as Co concentration increases, the band gap energy of the QDs also increases. Additionally, Co^2+^ substitutes Cd^2+^ lattice sites, causing strain, which alters the energy band structure and pushes the conduction band edge higher, leading to a wider band gap. However, E. Bacaksiz *et al.* indicated that the band gap energy decreases with increasing Co concentration.^32^

**Table 3:** Abs peak, PL peaks, QY, and bangap energy of CdSSe doped Co

Sample	Abs peak (nm)	PL peak (nm)	QY (%)	*E*_g_ (eV)
CdSSe	468	552	43.36	2.49
CdSSe:Co 1%	461	521	25.26	2.52
CdSSe:Co 3%	437	518	16.55	2.63
CdSSe:Co 5%	423	499	14.87	2.69
CdSSe:Co 10%	395	478	8.25	2.84

#### 3.2.3 Structural and elemental analysis

**Fig. 10:**
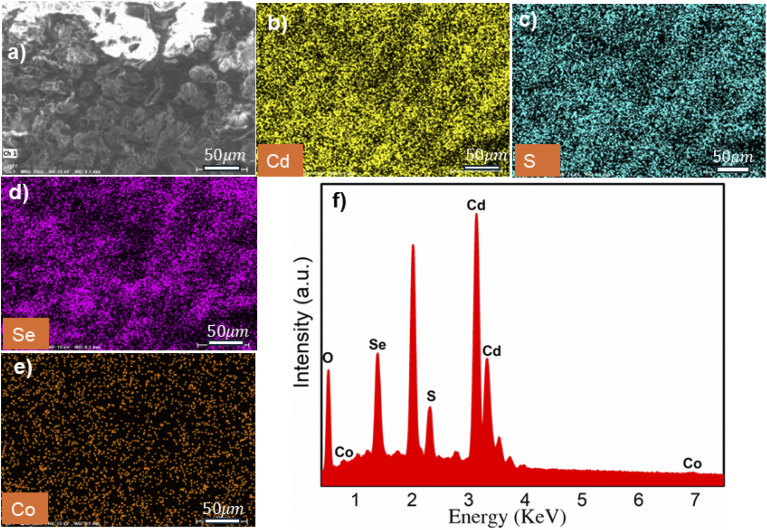
(a) EDX elemental mapping of CdSSe doped Co 3% for (b) cadmium, (c) sulfur, (d) selenium and (e) cobalt; (f) the corresponding EDX spectra of the as-synthesized CdSSe doped 3%.

EDX spectra determine the presence and ratio of elements in the samples. Fig. 10 displays the EDX spectrum and EDX mapping of 3% Co-doped CdSSe QDs. EDX mapping in Fig. 10a–e reveals the presence of Cd, S, Se, and Co elements in the sample, with uniform distribution. This demonstrates the successful synthesis of 3% Co-doped CdSSe QDs. Fig. 10f shows strong characteristic peaks at energies 3.1 keV, 2.3 keV, and 1.38 keV, corresponding to the elements Cd, S, and Se. Additionally, characteristic peaks at 0.78 keV and 6.9 keV are typical for the element Co. The presence of O and P elements in the EDX spectrum can be attributed to the residual ligands and solvents attached to the QDs surface. This result further confirms the successful synthesis of CdSSe:Co 3% QDs. Table 4 presents the elemental analysis results of CdSSe:Co 1% QDs, CdSSe:Co 3% QDs, and CdSSe:Co 5% QDs. As Co concentration increased, the chemical composition of Cd tended to increase from 46.23% to 62.08%, while the chemical composition of S significantly decreased from 23.57% to 11.46%. In contrast, the chemical composition of Se varies within the range of 20–30%. Meanwhile, the S content decreases significantly, which is attributed to the formation of sulfur vacancies. These vacancies play a crucial role in influencing the optical and electronic properties of the material system. This result is consistent with previous studies on transition metal ion-doped CdS QDs, where Co doping led to the modification of surface chemical composition and lattice structure. The actual concentrations of the Co element determined from EDX spectra were 0.14, 2, 6.88%, corresponding to theoretical concentrations of 1, 3, and 5%. The actual Co/Cd elemental ratio is lower than the theoretical value, indicating that a considerable number of Co ions do not substitute Cd ions and are removed during the sample purification process.

**Table 4:** Weight and atomic percentages of doped and undoped QDsdetermined from EDX measurements

Sample	Atomic (%)				Weight (%)			
	Cd	S	Se	Co	Cd	S	Se	Co
CdSSe	46.23	23.57	30.2	—	62.33	9.07	28.61	—
CdSSe:Co 1%	59.95	11.46	28.46	0.14	71.99	3.92	24	0.09
CdSSe:Co 3%	62.08	15.11	20.81	2	75.65	5.25	17.82	1.28
CdSSe:Co 5%	56.18	12.75	24.19	6.88	69.86	4.52	21.13	4.48

**Fig. 11:**
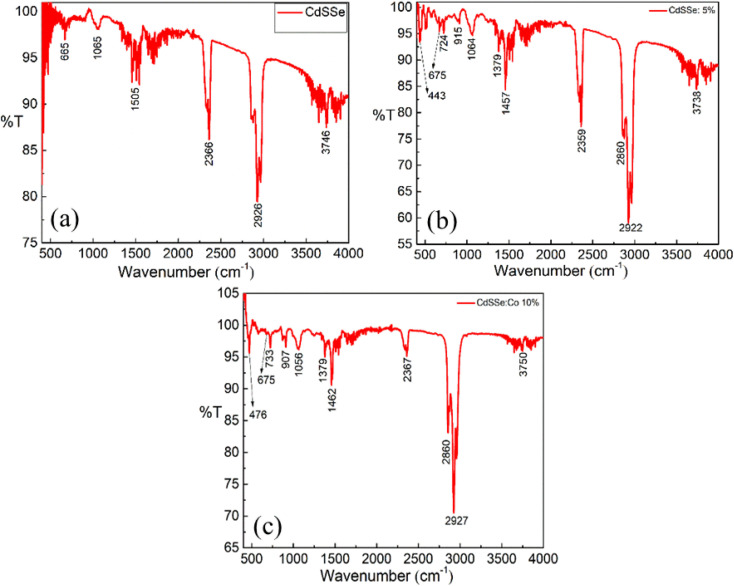
FTIR spectra of undoped and Co doped CdSS QDs. (a) CdSSe, (b) 5% Co doped CdSSe, (c) 10% Co doped CdSSe.

Fig. 11 presents the FTIR spectra of pure CdSSe QDs and Co-doped CdSSe QDs with doping concentrations of 5% and 10%, measured at room temperature over the wavenumber range of 400–4000 cm^−1^. The absorption bands corresponding to the vibrational and stretching modes of the functional groups in the samples, as well as the IR transmittance values, are listed in Table 5. The appearance of the Co–S stretching vibration band confirms the incorporation of Co into the crystal lattice of CdSSe:Co QDs, whereas this band is absent in the undoped sample.^25,33,34^

**Table 5:** Functional group of undoped and Co doped CdSSe QDs

Wave no. (cm^−1^)			Transmittance (%)			Functional group
CdSSe	5% Co	10% Co	CdSSe	5% Co	10% Co	
—	443	476	—	93	95.5	Co–S stretching mode
665	675	675	96	95	98	Cd–S stretching mode
731	724	733	98	94	96	O–O stretching mode
—	915	907	95	96	96.5	
1065	1064	1056	98	94	95.9	
1391	1379	1379	95	91	94.7	
1505	1457	1462	92	84	90.5	C–C stretching mode
2366	2359	2367	86	77	95	
2879	2860	2860	88	75	83	Symmetric C–H stretching mode
2926	2922	2927	79	59	70	Asymmetric C–H stretching mode
3746	3738	3750	87	84	95	

#### 3.2.4 Photoluminescence decay curves

**Fig. 12:**
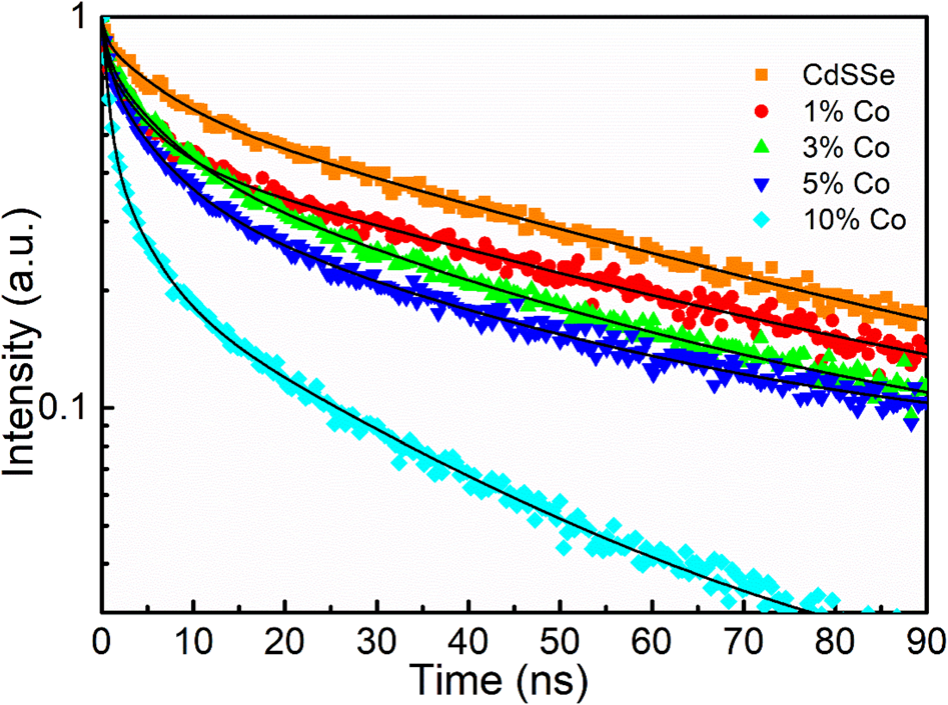
PL decay kinetics recorded at *E*_peak_ of representative CdSSe:Co QDs.

Fig. 12 presents the time-resolved photoluminescence spectra of CdSSe QDs and Co-doped CdSSe QDs with doping concentrations of 1, 3, 5, 10%, under excitation at 375 nm. The fluorescence decay curves of the samples followed a multi-exponential function. These curves were fitted according to the following equation:^35,36^5
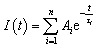
where *n* is the number of components contributed to the decay process; *A*_*i*_ and *τ*_*i*_ are the magnitude and lifetime of the ith component, respectively. The value of *τ*_*i*_ depends on the structure, elemental composition, size, and shape of the QDs. The average lifetime 〈*τ*〉 is determined from the expression:^37,38^6
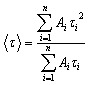
where *A*_*i*_ and *τ*_*i*_ (*i* = 1, 2, 3) represent the amplitude and the corresponding lifetime components, respectively. The shortest lifetime component (*τ*_1_) is generally associated with fast non-radiative recombination processes related to surface defects or trap states. The intermediate component (*τ*_2_) is attributed to carrier trapping and defect-assisted recombination within the host lattice. In contrast, the longest lifetime component (*τ*_3_) is typically assigned to radiative recombination of electron–hole pairs or excitons near the band edge. The relative amplitudes *A*_1_, *A*_2_, and *A*_3_ indicate the contribution of each recombination pathway to the overall PL decay behavior. The lifetime of undoped CdSSe QDs is 43.88 ns. As the concentration of Co doped into the CdSe host lattice increases, the lifetime gradually decreases from 31.11 ns (CdSSe:Co 1%) to 29.71 ns (CdSSe:Co 3%), 21.28 ns (CdSSe:Co 5%), and reaches a minimum value of 9.24 ns with CdSSe:Co 10% (Table 6). This decrease in lifetime is attributed to the formation of non-radiative centers upon Co doping into the host lattice.^39^ Specifically, the d energy levels of Co^2+^ ions can act as trap states, increasing the probability of non-radiative recombination and thereby shortening the lifetime of the excited state. In semiconductor materials, the photoluminescence lifetime *τ* is determined by the combined contributions of radiative and non-radiative recombination processes, which can be expressed as:

7 1/*τ* = *k*_r_ + *k*_nr_

where *k*_r_ and *k*_nr_ represent the radiative and non-radiative recombination rates, respectively. Upon Co doping, the introduced Co ions tend to generate defect levels or trap states within the band gap of the host lattice. These defect states act as additional non-radiative recombination centers, increasing the probability of energy dissipation through phonon-assisted processes. As a result, the non-radiative recombination rate *k*_nr_ increases with increasing Co concentration. According to the above relationship, an increase in *k*_nr_ leads to an increase in the total recombination rate (1/*τ*), thereby shortening the photoluminescence lifetime. Consequently, the observed decrease in lifetime with increasing Co concentration can be attributed to the enhanced non-radiative recombination induced by Co-related defect states.

**Table 6:** *τ*_*i*_, *A*_*i*_*,* and 〈*τ*〉 values for CdSSe:Co QDs with all doping ratios

Sample	*A*_1_	*τ*_1_ (ns)	*A*_2_	*τ*_2_ (ns)	*A*_3_	*τ*_3_ (ns)	〈*τ*〉 (ns)
CdSSe	0.27	6.43	0.6	46.85	0.13	3.52	43.88
CdSSe:Co 1%	0.32	5.32	0.53	34.28	0.15	3.27	31.11
CdSSe:Co 3%	0.33	5.09	0.52	32.86	0.15	3.12	29.71
CdSSe:Co 5%	0.41	4.57	0.42	24.94	0.17	2.18	21.28
CdSSe:Co 10%	0.52	3.02	0.29	12.43	0.19	1.12	9.24

Furthermore, the quantum yield (QY) of the CdSSe:Co QDs also decreases with increasing Co concentration, dropping from 43.36% for undoped CdSSe QDs to 25.26%, 16.55%, 14.87%, and 8.25% for the CdSSe:Co samples with 1, 3, 5, and 10% Co, respectively (Table 3). The simultaneous reduction in both QY and lifetime provides clear evidence for the enhancement of non-radiative recombination channels within the material system.

Mechanistically, when Co^2+^ ions substitutionally replace Cd^2+^ ions within the CdSSe crystal lattice, they introduce energy levels located within the bandgap of the host material. These energy levels act as carrier traps, effectively capturing electrons from the conduction band or holes from the valence band. At these trap sites, rather than undergoing radiative recombination to emit photons, the exciton energy is dissipated as thermal energy through exciton-phonon interactions. As the Co concentration increases, the density of these trap centers rises accordingly. This enhances the probability of charge carriers being captured at non-radiative centers prior to radiative recombination, consequently shortening the lifetime and diminishing the photoluminescence efficiency of the QDs.

### 3.3 The influence of Co on the magnetic properties of CdSSe QDs

**Fig. 13:**
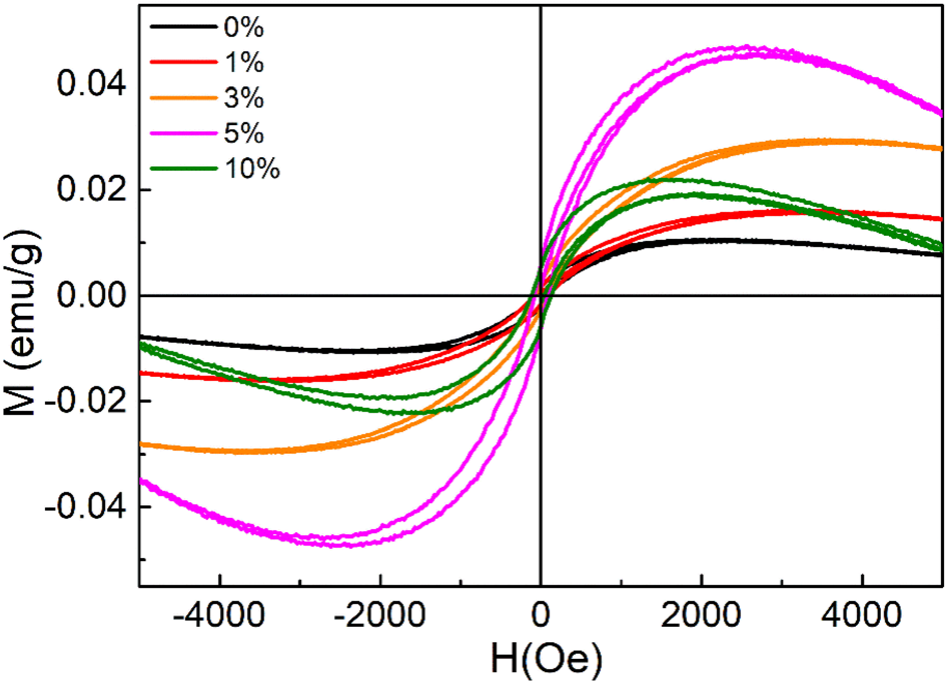
Room temperature M–H plots of CdSSe:Co (0, 1, 3, 5 and 10% Co) QDs.

The magnetic properties of CdSSe and Co-doped CdSSe QDs were investigated at room temperature using magnetization measurements under an applied magnetic field (M–H curves). Fig. 13 presents the hysteresis (M–H) curves of pure CdSSe QDs and Co-doped CdSSe QDs with Co concentrations ranging from 0 to 10%. All samples exhibit symmetric, nonlinear S-shaped magnetization curves around the origin, indicating a weak magnetic response at room temperature. However, a clear magnetic saturation is not observed within the applied field range of ±5000 Oe. Instead, the magnetization initially increases with the applied magnetic field, reaches a broad maximum, and then slightly decreases at higher fields. This behavior suggests that the total magnetic signal arises from a combination of different magnetic contributions rather than a purely saturated ferromagnetic phase. In particular, the overall magnetization may include a nonlinear magnetic component associated with Co-related magnetic centers together with an additional linear background contribution, possibly originating from the intrinsic diamagnetism of the semiconductor host or the sample environment.

For the undoped CdSSe QDs, the magnetization is very small, and the curve is relatively narrow, indicating that the pure CdSSe QDs are essentially nonmagnetic at room temperature. The weak magnetic signal observed in this sample is likely related to intrinsic structural defects, such as vacancies or surface states, which are commonly present in semiconductor QDs and can induce localized magnetic moments. Upon introducing Co dopants, the magnetic response becomes progressively stronger. For the samples doped with 1% and 3% Co, the magnetization increases noticeably, indicating that the incorporation of Co ions introduces additional localized magnetic moments into the system. These magnetic ions can interact with carriers or defect states in the semiconductor lattice through sp–d exchange interactions, leading to an enhanced magnetic response.

A further increase in the Co concentration to 5% results in the largest magnetization among all samples. This suggests that 5% Co represents an optimal doping level where the density of magnetic centers is sufficiently high to promote effective magnetic interactions without causing significant spin compensation. At this concentration, the average distance between Co ions becomes small enough to facilitate indirect exchange interactions mediated by anions (S/Se), carriers, or defect states within the CdSSe lattice. As a result, a stronger collective magnetic response can be observed, leading to the highest magnetization amplitude in the measured field range. Similar behavior has been reported in many diluted magnetic semiconductor nanostructures, where an intermediate dopant concentration maximizes the magnetic response due to an optimal balance between magnetic ion density and spin ordering.

Interestingly, when the Co concentration increases further to 10%, the magnetization decreases rather than continuing to increase. This reduction can be attributed to the onset of competing magnetic interactions at higher dopant concentrations. When the density of Co ions becomes too large, the probability of direct Co–Co interactions increases, which may lead to antiferromagnetic coupling between neighboring magnetic ions.^40,41^ Such interactions partially cancel the magnetic moments and therefore reduce the overall magnetization. In addition, higher dopant concentrations may also promote local clustering of Co ions or increased spin disorder, particularly at the surfaces of the QDs where coordination symmetry is reduced. These effects can further weaken the net magnetic response.^41,42^

Another notable feature of the M–H curves is that the magnetization increases rapidly in the low-field region but gradually decreases at higher magnetic fields. The initial increase in magnetization can be attributed to the progressive alignment of magnetic moments with the external magnetic field. In the absence of an applied field, the magnetic moments associated with Co ions and defect centers are randomly oriented. As the magnetic field increases, these moments tend to align along the field direction, leading to a rapid increase in magnetization. However, once most of the easily alignable moments become oriented, the nonlinear magnetic component approaches a quasi-saturation state. At higher fields, the linear background contribution—likely associated with the intrinsic diamagnetic response of the CdSSe host or other nonmagnetic components—becomes more pronounced. Since this contribution is opposite in sign to the main magnetic signal, it can cause the measured magnetization to decrease slightly at large fields.

## 4 Conclusion

In summary, Co-doped CdSSe QDs were successfully synthesized *via* a hot-injection method, and the effects of S/Se composition and Co^2+^ doping concentration on their structural, optical, and magnetic properties were systematically investigated. The results confirm that CdS_*x*_Se_1−*x*_ QDs crystallize in the cubic zinc blende structure with crystallite sizes ranging from 1.29 to 1.96 nm, while the band gap energy can be tuned from 3.2 eV to 2.2 eV by varying the S/Se ratio, resulting in photoluminescence emission shifting from 410 nm to 610 nm across the visible region. Upon Co doping (1–10%), the lattice constant decreases from 5.93 Å to 5.80 Å, indicating the successful substitution of Cd^2+^ ions by smaller Co^2+^ ions in the CdSSe lattice. Optical measurements reveal a pronounced blue shift in both absorption and photoluminescence spectra, where the absorption peak shifts from 468 nm to 395 nm, and the emission peak shifts from 552 nm to 478 nm, accompanied by an increase in band gap energy from 2.49 eV to 2.84 eV. In addition, the photoluminescence lifetime decreases significantly from 43.88 ns (undoped CdSSe) to 9.24 ns (CdSSe:Co 10%), while the quantum yield decreases from 43.36% to 8.25%, indicating enhanced non-radiative recombination through Co-induced trap states. Magnetic measurements demonstrate that Co doping induces weak room-temperature ferromagnetism in CdSSe QDs. The magnetic response increases with Co concentration and reaches a maximum at 5% Co doping, followed by a decrease at higher concentrations due to competing antiferromagnetic interactions between neighboring Co ions. These results demonstrate that Co incorporation provides an effective strategy to simultaneously tune the structural, optical, and magnetic properties of CdSSe quantum dots. Owing to their tunable band gap, composition-dependent luminescence, and room-temperature magnetic response, Co-doped CdSSe QDs are promising multifunctional materials for potential applications in optoelectronic devices, spintronic systems, photodetectors, and quantum-dot-based light-emitting devices.

## Conflicts of interest

There are no conflicts to declare.

## Data Availability

The data supporting this study's findings are available on request from the corresponding authors [Nguyen Xuan Ca, email: nguyenxuanca@tnus.edu.vn and Pham Minh Tan, email: tanpm@ptit.edu.vn]. The data are not publicly available due to privacy reasons.
